# Frequency and Geographic Distribution of *CARD9* Mutations in Patients With Severe Fungal Infections

**DOI:** 10.3389/fmicb.2018.02434

**Published:** 2018-10-12

**Authors:** Afsane Vaezi, Hamed Fakhim, Zahra Abtahian, Sadegh Khodavaisy, Mohsen Geramishoar, Ahad Alizadeh, Jacques F. Meis, Hamid Badali

**Affiliations:** ^1^Department of Medical Mycology, School of Medicine, Mazandaran University of Medical Sciences, Sari, Iran; ^2^Department of Medical Parasitology and Mycology, Faculty of Medicine, Urmia University of Medical Sciences, Urmia, Iran; ^3^Cellular and Molecular Research Center, Urmia University of Medical Sciences, Urmia, Iran; ^4^Infectious Disease and Tropical Medicine Research Center, Shahid Beheshti University of Medical Sciences, Tehran, Iran; ^5^Department of Medical Parasitology and Mycology, School of Public Health, Tehran University of Medical Sciences, Tehran, Iran; ^6^Department of Epidemiology and Reproductive Health, Reproductive Epidemiology Research Center, Royan Institute for Reproductive Biomedicine, Academic Center for Education, Culture and Research, Tehran, Iran; ^7^Department of Medical Microbiology and Infectious Diseases, ECMM Excellence Center for Medical Mycology, Canisius-Wilhelmina Hospital, Nijmegen, Netherlands; ^8^Centre of Expertise in Mycology Radboudumc/CWZ, Nijmegen, Netherlands; ^9^Invasive Fungi Research Center, School of Medicine, Mazandaran University of Medical Sciences, Sari, Iran

**Keywords:** severe fungal infections, CARD9 deficiency, mutation, candidiasis, dermatophytosis

## Abstract

Autosomal recessive deficiency in the caspase recruitment domain containing protein 9 (CARD9) results in susceptibility to fungal infections. In the last decade, infections associated with CARD9 deficiency are more reported due to the advent of genome sequencing. The aim of this study was to evaluate the frequency, geographic distribution and nature of mutations in patients with CARD9 deficiency. We identified 60 patients with 24 mutations and different fungal infections. The presence of the homozygous (HMZ) p.Q295X (c.883C > T) and HMZ p.Q289X (c.865C > T) mutations were associated with an elevated risk of candidiasis (OR: 1.6; 95% CI: 1.18–2.15; *p* = 0.004) and dermatophytosis (OR: 1.85; 95% CI: 1.47–2.37; *p* < 0.001), respectively. The geographical distribution differed, showing that the main mutations in African patients were different Asian patients; HMZ p.Q289X (c.865C > T) and HMZ p.Q295X (c.865C > T) accounted for 75% and 37.9% of the African and Asian cases, respectively. The spectrum of *CARD9* mutations in Asian patients was higher than in African. Asia is the most populous continent in the world and may have a greater genetic burden resulting in more patients with severe fungal infections. The presence of a high diversity of mutations revealing 24 distinct variations among 60 patients emphasize that the unique genetic alteration in *CARD9* gene may be associated with certain geographical areas.

## Introduction

Susceptibility to fungal infections in otherwise healthy individuals with Mendelian disorders are increasingly being recognized (Vinh, 2011) than before the widespread use of genome sequencing. Primary immunodeficiencies consist of various genetic defects that affect the innate and adaptive immune systems. In addition, evaluation of previously healthy, fungus infected patients, suspected of having a primary genetic immunodeficiency may give valuable insights on the role of specific proteins in the immune system for protection from these infections (Wang et al., 2014; Corvilain et al., 2018). Caspase recruitment domain containing protein 9 (CARD9) is a central regulator of innate immunity that is highly expressed in neutrophils, macrophages, dendritic cells, and during cell apoptosis in low-serum conditions (Bertin et al., 2000; Liang et al., 2015). Mutations in several proteins involved in the CARD9 signaling protein have been demonstrated to cause primary immunodeficiencies in humans. These mutations cause a decreased production of cytokines from innate immune cells, leading to deficiencies of TH17 and accordingly predispose patients to severe disseminated infections (Conti and Gaffen, 2015). Severe fungal infections in healthy patients have recently been reported from a few countries, i.e., Algeria, Brazil, France, China, Iran, Morocco and Tunisia (Glocker et al., 2009; Drewniak et al., 2013; Lanternier et al., 2013; Wang et al., 2014; Grumach et al., 2015) and linked to autosomal recessive CARD9 deficiency. The species involved in these infections are *Trichophyton violaceum, Trichophyton rubrum*, *Candida* species, *Exophiala* species, *Phialophora verrucosa*, *Aspergillus* *fumigatus, Prototheca* *zopfii, and Mucor irregularis.* Some of those etiological agents are plant pathogens, which rarely have been associated with human infection. Highly diverse clinical manifestations from cutaneous to disseminated and progressive infections are observed (Boudghène-Stambouli and Mérad-Boudia, 1991; Boudghène-Stambouli et al., 1992; Pruszkowski et al., 1995). Our aim was to evaluate the global frequency, geographic distribution and nature of mutations in patients with CARD9 deficiency associated with fungal infections.

## Materials and Methods

The review process involved study of existing published literature of all reported cases with fungal infection due to CARD9 deficiency. To search the published literature, Medline database through PubMed, Embase through Scopus, ISI Web of Science, Science Direct and Google Scholar were used to explore the published literature of patients with severe fungal infection and CARD9 deficiency using the key words “caspase recruitment domain deficiency,” “CARD9 deficiency,” “autosomal recessive CARD9 deficiency,” “primary immunodeficiency,” “mutations,” “fungal infection” or “invasive fungal diseases,” “candidiasis,” “deep dermatophytosis,” “disseminated phaeohyphomycosis,” and “chronic mucocutaneous candidiasis” in different combinations. A total of 21 relevant articles were found using these key words. The extracted data were analyzed using R software version 3.4.1. The chi-square test was utilized to evaluate associations between nominal variables and the *p*-value was estimated using the Monte Carlo method. To compare the differential prevalence of *CARD9* mutations and determine differences in causative agents of fungal infections, odds ratios (ORs) were used. The significance of all ORs, using a 95% Bayesian credible interval (CI), was calculated using Bayesian logistic regression.

## Results

### The Burden of CARD9 Deficiency Is Positively Correlated With Fungal Infection

**FIGURE 1:**
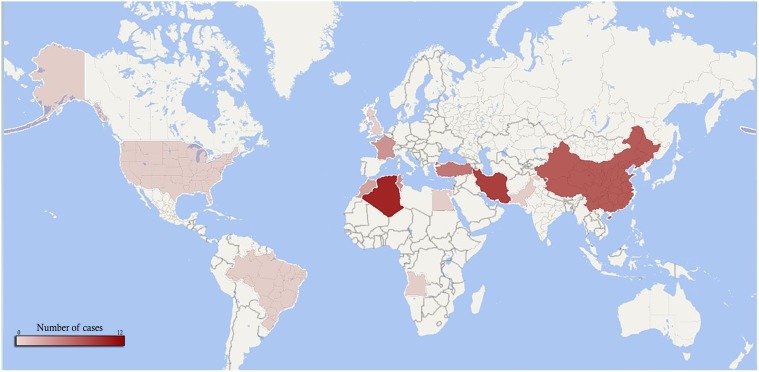
The worldwide distributions of fungal infection cases with CARD9 deficiency.

To analyze the role of CARD9 deficiency in fungal infection, we reviewed the literature and identified 60 cases until 2018. The total number of patients with severe fungal infection related to CARD9 deficiency has been summarized in **Tables 1A**,**B** (Boudghène-Stambouli and Mérad-Boudia, 1989, 1991, 1998; Pruszkowski et al., 1995; Glocker et al., 2009; Drewniak et al., 2013; Gavino et al., 2014; Wang et al., 2014; Drummond et al., 2015; Grumach et al., 2015; Herbst et al., 2015; Jachiet et al., 2015; Lanternier et al., 2015a,b; Alves de Medeiros et al., 2016; Gavino et al., 2016; Jones et al., 2016; Rieber et al., 2016; Yan et al., 2016; Boudghene-Stambouli et al., 2017; Gavino et al., 2018; Sari et al., 2018; Vaezi et al., 2018; Wang et al., 2018a,b). The age at the time of diagnosis ranged from 4 to 91 years (mean 34.3 ± 17.9 years). Since 1989, a total of 14 countries reported cases of fungal infections associated with CARD9 deficiency (**Figure 1**). Although most cases originate from Algeria (North Africa) [*n* = 12 (21.1%)], the majority of cases were from several countries in the Asian continent (*n* = 29, 48.3%), with Iran reporting the majority (*n* = 10/29, 34.5%). The main fungal infection associated with CARD9 deficiency was candidiasis (40.3%) followed by deep dermatophytosis (37.3%), phaeohyphomycosis (16.4%) and invasive aspergillosis (3.0%). *T. violaceum*, *T. rubrum*, and *Trichophyton mentagrophytes* were observed as etiological agents of dermatophytosis. *Candida* infections were caused by *C. albicans* and non-*albicans Candida* species in 70.8% and 29.2% of the cases, respectively. *P. verrucosa* (36.4%) represented the major species of phaeohyphomycosis and were only reported from China. Neurological infection (40.5%) was the predominant clinical presentation in *Candida* infected patients followed by chronic mucosal and cutaneous candidiasis (29.7%). The outcome was recorded in 45 cases and 11 (24.4%) expired.

**Table 1A:** Prevalence of fungal infections, duration of infections and causative pathogens in patients with CARD9 deficiency.

Fungal infection	Duration of infection, mean ( ± SD), year	Nr of cases (%)	Causative agent	Nr of cases (%)
Dermatophytosis	37.8 ± 18.7	25 (37.3)	*Trichophyton rubrum*	7 (13.0)
Phaeohyphomycosis	8.5 ± 6.6	11 (16.4)	*Trichophyton violaceum*	8 (14.8)
Invasive aspergillosis	-	2 (3.0)	*Trichophyton mentagrophytes*	1 (1.9)
Mucormycosis	-	1 (1.5)	*Candida* spp	5 (9.3)
Protothecosis	-	1 (1.5)	*Candida albicans*	17 (31.5)
Candidiasis	8.5 ± 10.8	27 (40.3)	*Candida dubliniensis*	1 (1.9)
Mucosal and cutaneous candidiasis	11.5 ± 15.5	11 (29.7)	*Candida glabrata*	1 (1.9)
Neurologic infection	5.3 ± 5.6	15 (40.5)	*Phialophora verrucosa*	4 (7.4)
Chronic candidiasis	6.5 ± 7.7	4 (10.8)	*Exophiala dermatitidis*	1 (1.9)
Osteomyelitis	3.3 ± 0.5	3 (8.1)	*Exophiala spinifera*	2 (3.7)
Endophthalmitis	2.3 ± 1.1	3 (8.1)	*Aspergillus fumigatus*	2 (3.7)
Colitis	-	1 (2.7)	*Corynespora cassiicola*	2 (3.7)
			*Ochroconis musae*	1 (1.9)
			*Mucor irregularis*	1 (1.9)
			*Prototheca zopfii*	1 (1.9)


*Neurologic infection includes meningoencephalitis, meningitis, and brain abscesses.*

**Table 1B:** Overview of patient demographics and mutations.

Condition	Nr of cases (%)	Mutation	Nucleotide change	Domain	Nr of cases (%)
**Age (year)**		HMZ Q289X	c.865C>T	CCD	16 (25.8)
<20	16 (26.7)	HMZ Q295X	c.883C>T	CCD	11 (17.7)
21–60	39 (65)	HMZ D274fsX60	c.819-820insG	CCD	5 (8.1)
>60	5 (8.3)	HMZ R70W	c.208C>T	CARD	4 (6.5)
**Male/female**	30(50)/30(50)	HMZ Y91H	c.271T>C	CARD	4 (6.5)
**Country**		HTZ L64fsX59	c.191–192insTGCT	CARD	3 (4.8)
Algeria	12 (21.1)	HMZ R101C	c.C301T	CARD	2 (3.2)
Angola	1 (1.7)	HTZ Q158X	c.472C>T	CCD	1 (1.6)
Brazil	1 (1.7)	HTZ G72S	c.214G>A	CARD	1 (1.6)
China	9 (15.8)	HTZ R373P	c.1118G>C	CCD	1 (1.6)
Egypt	1 (1.7)	HMZ R35Q	c.104G>A	CARD	1 (1.6)
France	4 (7.0)	HMZ R18W	c.52C>T	CARD	1 (1.6)
Iran	10 (17.5)	HMZ E323del	c.GAG967-969del	CCD	1 (1.6)
Korea	1 (1.7)	HMZ R101L	c.302G>T	CARD	1 (1.6)
Morocco	3 (5.3)	HMZ R57H	c.170G>A	CARD	1 (1.6)
Pakistan	1 (1.7)	HMZ M1I	c.3G>C	CARD	1 (1.6)
Tunisia	4 (7.0)	HTZ A380P	c.1138G>C	CCD	1 (1.6)
Turkey	8 (14.0)	HTZ R317R	c.951G>A	CCD	1 (1.6)
United Kingdom	1 (1.7)	HTZ S23X	c.68C>A	CARD	1 (1.6)
United States	1 (1.7)	HMZ V261fs	c.781delG	CCD	1 (1.6)
		HTZ G62fs	c.184G>A	CARD	1 (1.6)
		HTZ G96del36	c.288C>T	CARD	1 (1.6)
		HTZ T231M	c.692C>T	CCD	1 (1.6)
		HTZ F302del	c.905_907delTCT	CCD	1 (1.6)


*HMZ, homozygous; HTZ, heterozygous; CCD, coiled-coiled domain of CARD9 protein; CARD, CARD domain of CARD9 protein.*

### Associations Among Mutations of the *CARD9* Gene and Infection Status With Fungal Pathogens

**FIGURE 2:**
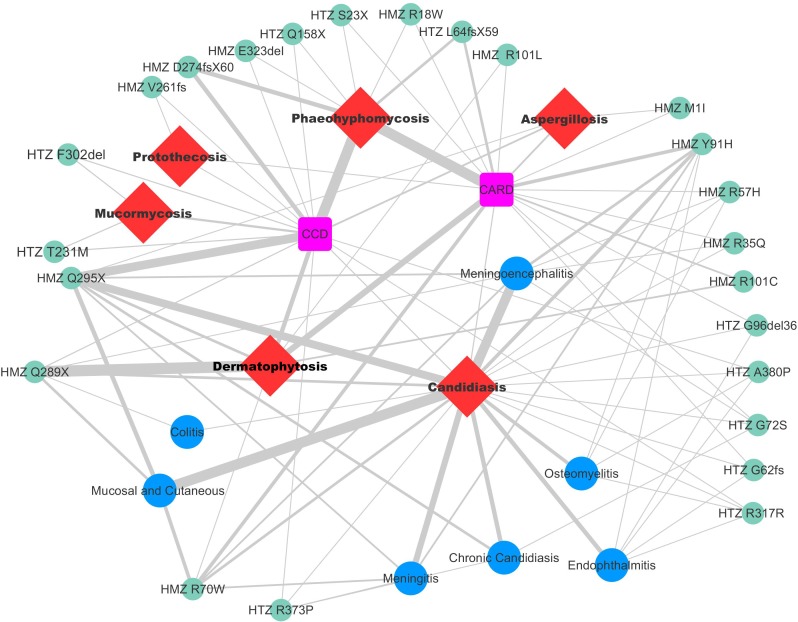
Relation between the types of mutations and different clinical forms. Thick, short, and bold line indicates more reported cases with a mutation and clinical form; and less cases shown with thin and lane lines.

Overall, 24 different genetic alterations in *CARD9* were described in the 60 patients. Three of those were identified most frequently: homozygous (HMZ) p.Q289X (c.865C > T), HMZ p.Q295X (c.883C > T) and HMZ p.D274fsX60 (c.819-820insG), which accounted for 25.8%, 17.7%, and 8.1% of the patients, respectively. Multiple variations in *CARD9* were identified in 8.7% of all cases. The correlation between mutations and fungal infection is shown in **Figure 2**. The presence of the HMZ p.Q295X (c.883C > T) and HMZ p.Q289X (c.865C > T) mutation was associated with an elevated risk of candidiasis (OR: 1.6; 95% CI: 1.18–2.15; *p* = 0.004) and dermatophytosis (OR: 1.85; 95% CI: 1.47–2.37; *p* < 0.001), respectively. Also a strong association was evident between the presence of HMZ p.D274fsX60 (c.819-820insG) and disseminated phaeohyphomycosis; 2.42 (95% CI 1.84–3.2, *p* < 0.001). This study demonstrated that the HMZ p.Q289X (c.865C > T) mutation had a more than two-fold increased risk of dermatophytosis compared with HMZ p.Q295X (c.883C > T), *p* < 0.001. Similarly, HMZ p.Q295X (c.883C > T) alteration increased by two times the risk of developing candidiasis [OR: 1.95 (95% CI 1.42-2.69, *p* < 0⋅001)] versus dermatophytosis (**Table 2**). *T. violaceum* infected patients carried a marginally higher frequency of HMZ p.Q289X (c.865C > T) compared to non-*T. violaceum* dermatophytosis cases (43 vs. 56k%).

**Table 2:** Analysis of 24 reported mutations among 60 patients with fungal infections.

			Dermatophytosis		Phaeohyphomycosis		Invasive aspergillosis		Candidiasis		Mucormycosis		Protothecosis	

Model type	Factor		OR (95% CI)	*P*-value	OR (95% CI)	*P*-value	OR (95% CI)	*P-*value	OR (95% CI)	*P*-value	OR (95% CI)	*P*-value	OR (95% CI)	*P*-value
Crude analysis	Mutation	HMZ p.Q289X^∗^	1	-	1	-	1	-	1	-	1	-	1	-
		HMZ p.Q295X	0.53 (0.42–0.67)	<0.001	1.09 (0.84–1.43)	0.506	1.09 (0.96–1.25)	0.19	1.95 (1.41–2.67)	<0.001	1 (0.91–1.1)	0.998	1 (0.91–1.1)	0.998
		Other	0.44 (0.36–0.52)	<0.001	1.45 (1.18–1.78)	0.001	1.04 (0.93–1.15)	0.494	1.4 (1.09–1.78)	0.012	1.04 (0.96–1.12)	0.337	1.04 (0.96–1.12)	0.337
	Domain	CCD^∗^	1	-	1	-	1	-	1	-	1	-	1	-
		CARD	0.64 (0.5–0.83)	0.001	1.11 (0.89–1.38)	0.342	1.03 (0.93–1.15)	0.54	1.37 (1.04–1.81)	0.031	0.98 (0.9–1.05)	0.509	0.97 (0.9–1.05)	0.509
														
Multivariate analysis^∗∗^	Mutation	HMZ p.Q289X^∗^	1	-	1	-	1	-	1	-	1	-	1	-
		HMZ p.Q295X	0.52 (0.4–0.66)	<0.001	1.15 (0.84–1.57)	0.412	1.11 (0.94–1.3)	0.197	2.09 (1.43–3.07)	<0.001	1.02 (0.91–1.15)	0.728	0.96 (0.85–1.08)	0.477
		Other	0.48 (0.4–0.58)	<0.001	1.45 (1.15–1.83)	0.003	1.05 (0.93–1.18)	0.429	1.4 (1.06–1.85)	0.022	1.05 (0.96–1.14)	0.294	1 (0.92–1.09)	0.908
	Domain	CCD^∗^	1	-	1	-	1	-	1	-	1	-	1	-
		CARD	0.72 (0.57–0.91)	0.007	1.09 (0.87–1.36)	0.468	1.03 (0.93–1.15)	0.582	1.33 (1–1.76)	0.053	0.97 (0.9–1.05)	0.486	0.96 (0.89–1.04)	0.305


*Levels; ^∗∗^The results were adjusted for age and sex; ^∗^CCD and HMZ p.Q289X were reference levels in their categories; OR, Odds ratio; CI, Bayesian credible interval; HMZ, homozygote; CCD, coiled-coiled domain; CARD, CARD domain; Mutations in CCD, Q289X, Q295X, R373P, Q158X, D274fsX60, E323del, A380P, R317R, V261fs, T231M, F302del; Mutations in CARD, Y91H, R70W, R35Q, R18W, G62fs, G96del36, R101C, G72S, L64fsX59, R101L, R57H, M11, S23X; Other mutations, Q289X, Q295X are not included.*

### A Relationship Between *CARD9* Gene Mutations and Specific Geographic Distribution

The pattern of distribution was differed by geographical region in reported cases with *CARD9* mutations. The main mutations in African patients were different from those in Asians; HMZ p.Q289X (c.865C > T) and HMZ p.R101C (c.C301T), accounting for 75% and 10%, respectively, were the common mutations in Africa. The three most common mutations in Asia were HMZ p.Q295X (c.883C > T), HMZ p.D274fsX60 (c.819-820insG), and HMZ p.R70W (c.208C > T), which accounted for 34.5%, 17.2%, and 13.8% of the Asian cases, respectively. Notably, HMZ p.Q289X (c.865C > T) was the most common mutation observed in 75% of the Algerian patients (9 out of 12), while the HMZ p.Q295X (c.883C > T) mutation was reported in 8 out of 10 Iranian patients (80%). This finding is important as it provides a relationship between mutation and specific geographic occurrence in these patients.

## Discussion

CARD9 deficiency is inherited in an autosomal recessive manner. CARD9 plays an important role in the activation of antifungal mechanisms leading to expression of gene products that initiate the inflammatory cascade (Liang et al., 2015; Drummond and Lionakis, 2016). The importance of the CARD9 signaling protein in host defense has been demonstrated in a murine CARD9-/- model with targeted disruptions of innate signaling from the antifungal pattern-recognition receptor, dectin-1, that identifies the β-glucan component of the fungal cell (Taylor et al., 2007). Defective antifungal clearance and latently infected cells could be the result of impaired CARD9 function (Yamamoto et al., 2014; Drummond and Lionakis, 2016). We analyzed the characteristics, distribution, frequency, and relationship between the genotype of the *CARD9* gene mutations and fungal infections among the reported cases. Since the first mutation described in 1989 from Algeria (Boudghène-Stambouli and Mérad-Boudia, 1989), several mutations have been reported from Africa. However, only few reports are from Europe and America. Glocker et al. (2009), reported a novel *CARD9* mutation, HMZ p.Q289X (c.883C > T), in seven Iranian patients. In this review, the spectrum of *CARD9* mutations in Asian patients is higher than in African patients. So far, more than 24 mutations in the *CARD9* gene have been reported associated with severe fungal infections. Among these mutations, HMZ p.Q289X (c.865C > T) was the most common, indicating it is a hot spot in Africa. Infections caused by *T. violaceum* and *C. albicans* dominate, but frequency differ by region. We found a remarkably low prevalence of dermatophyte infection in Asian CARD9 deficiency patients. However, we demonstrate that *Candida* species infection is also uncommon in African patients. Our review showed that the two mutations [HMZ p.Q289X (c.865C > T) and HMZ p.Q295X (c.883C > T)] are present in 44.3% of the patients. Dermatophytosis due to the HMZ p.Q289X (c.865C > T) mutation encompass 75% of African cases and 34.5% of Asian patients have candidiasis associated with HMZ p.Q295X (c.883C > T). However, mutations such as HMZ p.R57H (c.170G > A), heterozygous (HTZ) p.A380P (c.1138G > C) and HMZ p.R70W (c.208C > T) are only found in the United States, United Kingdom, and Turkey, respectively, which suggests that mutations may be specific in particular populations or geographic regions. Another possible explanation is the high rate of consanguinity in many closed groups. Although this autosomal recessive disorder which is rare on a world-wide scale, it may not be rare in some countries. The variations in the gene, which are associated with a specific fungal infection, remain unknown. Asia is the most populous continent in the world and may have a greater genetic burden resulting in more patients with severe fungal infections. Although we cannot exclude other causative factors, our data support the notion that some *CARD9* mutations, circulating in specific geographic regions, could be the contributing factor for fungal infections. However, because of the small sample size, future screening should be conducted to confirm these conclusions. Studying the impact of genetic variation on severe fungal infection will improve our understanding of pathogenesis and may ultimately aid future interventions. CARD9 deficiency should be considered in patients with unexplained progressive fungal infection, as it may allow early initiation of appropriate antifungal treatment. Regular medical follow-up and identification of patients with CARD9 deficiencies is recommended including family members.

## Conclusion

In recent years, interest in primary immunodeficiency disorders and opportunistic infections has grown. The current study reviewed 60 reported cases with *CARD9* mutations and severe fungal infections, which may provide more information about the relationship between these mutations, the specific geographic presence and the unique predisposition to a particular fungal disease.

## Author Contributions

AV, HB, and JM conceptualized the study, gathered resources, and wrote, reviewed, and edited the manuscript. AV, HF, ZA, MG, SK, and AA curated the data. AV, HB, and AA performed the formal analysis of the study. HB contributed to funding acquisition, project administration, and data validation. AV and HF investigated the data. AV, HF, ZA, MG, and SK provided methodology for this study. HB and JM supervised the study. AV, HF, ZA, MG, SK, AA, JM, and HB wrote the original draft of the manuscript.

## Conflict of Interest Statement

The authors declare that the research was conducted in the absence of any commercial or financial relationships that could be construed as a potential conflict of interest.

## References

[B1] Alves de Medeiros A. K. Lodewick E. Bogaert D. J. Haerynck F. Van Daele S. Lambrecht B. (2016). Chronic and invasive fungal infections in a family with CARD9 deficiency. *J. Clin. Immunol.* 36 204–209. 10.1007/s10875-016-0255-8 26961233

[B2] Bertin J. Guo Y. Wang L. Srinivasula S. M. Jacobson M. D. Poyet J. L. (2000). CARD9 is a novel caspase recruitment domain-containing protein that interacts with BCL10/CLAP and activates N-F-kappa B. *J. Biol. Chem.* 275 41082–41086. 10.1074/jbc.C000726200 11053425

[B3] Boudghene-Stambouli O. Amrani N. Boudghéne Stambouli K. Bouali F. (2017). Dermatophytic disease with deficit in CARD9: a new case with a brain impairment. *J. Mycol. Med.* 27 250–253. 10.1016/j.mycmed.2017.01.001 28391957

[B4] Boudghène-Stambouli O. Mérad-Boudia A. (1989). *Trichophyton rubrum* dermatophytic disease: a new case. *Ann. Dermatol. Venereol.* 116 725–727. 2610465

[B5] Boudghène-Stambouli O. Mérad-Boudia A. (1991). Dermatophytic disease in Algeria: a new case and review of the literature. *Ann. Dermatol. Venereol.* 118 17–21. 2018301

[B6] Boudghène-Stambouli O. Mérad-Boudia A. (1998). Dermatophytic disease: exuberant hyperkeratosis with cutaneous horns. *Ann. Dermatol. Venereol.* 125 705–707. 9835960

[B7] Boudghène-Stambouli O. Mérad-Boudia A. Allal M. (1992). Cerebral injury in dermatophytic disease. *J. Mycol. Med.* 2 106–108.

[B8] Conti H. R. Gaffen S. L. (2015). IL-17-mediated immunity to the opportunistic fungal pathogen *Candida albicans*. *J. Immunol.* 195 780–788. 10.4049/jimmunol.1500909 26188072 PMC4507294

[B9] Corvilain E. Casanova J. L. Puel A. (2018). Inherited CARD9 deficiency: invasive disease caused by Ascomycete fungi in previously healthy children and adults. *J. Clin. Immunol.* 38 656–693. 10.1007/s10875-018-0539-2 30136218 PMC6157734

[B10] Drewniak A. Gazendam R. P. Tool A. T. van Houdt M. Jansen M. H. van Hamme J. L. (2013). Invasive fungal infection and impaired neutrophil killing in human CARD9 deficiency. *Blood* 121 2385–2392. 10.1182/blood-2012-08-450551 23335372

[B11] Drummond R. A. Collar A. L. Swamydas M. Rodriguez C. A. Lim J. K. Mendez L. M. (2015). CARD9-dependent neutrophil recruitment protects against fungal invasion of the central nervous system. *PLoS Pathog.* 11:e1005293. 10.1371/journal.ppat.1005293 26679537 PMC4683065

[B12] Drummond R. A. Lionakis M. S. (2016). Mechanistic insights into the role of C-type lectin receptor/CARD9 signaling in human antifungal immunity. *Front. Cell. Infect. Microbiol.* 6:39. 10.3389/fcimb.2016.00039 27092298 PMC4820464

[B13] Gavino C. Cotter A. Lichtenstein D. Lejtenyi D. Fortin C. Legault C. (2014). CARD9 deficiency and spontaneous central nervous system candidiasis: complete clinical remission with GM-CSF therapy. *Clin. Infect. Dis.* 59 81–84. 10.1093/cid/ciu215 24704721 PMC4305130

[B14] Gavino C. Hamel N. Zeng J. B. Legault C. Guiot M. C. Chankowsky J. (2016). Impaired RASGRF1/ERK-mediated GM-CSF response characterizes CARD9 deficiency in French-Canadians. *J. Allergy Clin. Immunol.* 137 1178.e7–1188.e7. 10.1016/j.jaci.2015.09.016 26521038

[B15] Gavino C. Mellinghoff S. Cornely O. A. Landekic M. Le C. Langelier M. (2018). Novel bi-allelic splice mutations in *CARD9* causing adult-onset *Candida* endophthalmitis. *Mycoses* 61 61–65. 10.1111/myc.12701 28984994

[B16] Glocker E. O. Hennigs A. Nabavi M. Schaffer A. A. Woellner C. Salzer U. (2009). A homozygous *CARD9* mutation in a family with susceptibility to fungal infections. *N. Engl. J. Med.* 361 1727–1735. 10.1056/NEJMoa0810719 19864672 PMC2793117

[B17] Grumach A. S. de Queiroz-Telles F. Migaud M. Lanternier F. Filho N. R. Palma S. M. (2015). A homozygous *CARD9* mutation in a Brazilian patient with deep dermatophytosis. *J. Clin. Immunol.* 35 486–490. 10.1007/s10875-015-0170-4 26044242

[B18] Herbst M. Gazendam R. Reimnitz D. Sawalle-Belohradsky J. Groll A. Schlegel P. G. (2015). Chronic *Candida albicans* meningitis in a 4-year-old girl with a homozygous mutation in the *CARD9* gene (Q295X). *Pediatr. Infect. Dis. J.* 34 999–1002. 10.1097/INF.0000000000000736 25933095

[B19] Jachiet M. Lanternier F. Rybojad M. Bagot M. Ibrahim L. Casanova J. L. (2015). Posaconazole treatment of extensive skin and nail dermatophytosis due to autosomal recessive deficiency of CARD9. *JAMA Dermatol.* 151 192–194. 10.1001/jamadermatol.2014.2154 25372963

[B20] Jones N. Garcez T. Newman W. Denning D. (2016). Endogenous *Candida* endophthalmitis and osteomyelitis associated with CARD9 deficiency. *BMJ Case Rep.* 3:2016. 10.1136/bcr-2015-214117 26941346 PMC4785415

[B21] Lanternier F. Barbati E. Meinzer U. Liu L. Pedergnana V. Migaud M. (2015a). Inherited CARD9 deficiency in 2 unrelated patients with invasive *Exophiala* infection. *J. Infect. Dis.* 211 1241–1250. 10.1093/infdis/jiu412 25057046 PMC4447834

[B22] Lanternier F. Mahdaviani S. A. Barbati E. Chaussade H. Koumar Y. Levy R. (2015b). Inherited CARD9 deficiency in otherwise healthy children and adults with *Candida* species-induced meningoencephalitis, colitis, or both. *J. Allergy Clin. Immunol.* 135 1558.e2–1568.e2. 10.1016/j.jaci.2014.12.1930 25702837 PMC4831587

[B23] Lanternier F. Pathan S. Vincent Q. B. Liu L. Cypowyj S. Prando C. (2013). Deep dermatophytosis and inherited CARD9 deficiency. *N. Engl. J. Med.* 369 1704–1714. 10.1056/NEJMoa1208487 24131138 PMC4084693

[B24] Liang P. Wang X. Wang R. Wan Z. Han W. Li R. (2015). CARD9 deficiencies linked to impaired neutrophil functions against *Phialophora verrucosa*. *Mycopathologia* 179 347–357. 10.1007/s11046-015-9877-2 25790941

[B25] Pruszkowski A. Bourgault Villada I. Cremer G. Ammar-Khodja A. Emilie D. Revuz J. (1995). Dermatophytic disease: role of type TC2 CD8 lymphocytes. *Ann. Dermatol. Venereol.* 122(Suppl. 1) 55.

[B26] Rieber N. Gazendam R. P. Freeman A. F. Hsu A. P. Collar A. L. Sugui J. A. (2016). Extrapulmonary *Aspergillus* infection in patients with CARD9 deficiency. *JCI Insight* 1:e89890. 10.1172/jci.insight.89890 27777981 PMC5070961

[B27] Sari S. Dalgic B. Muehlenbachs A. DeLeon-Carnes M. Goldsmith C. S. Ekinci O. (2018). *Prototheca zopfii* colitis in inherited CARD9 deficiency. *J. Infect. Dis.* 218 485–489. 10.1093/infdis/jiy198 29659908 PMC6049027

[B28] Taylor P. R. Tsoni S. V. Willment J. A. Dennehy K. M. Rosas M. Findon H. (2007). Dectin-1 is required for beta-glucan recognition and control of fungal infection. *Nat. Immunol.* 8 31–38. 10.1038/ni1408 17159984 PMC1888731

[B36] Vaezi A. Mardani M. Fakhim H. Hedayat Yaghoobi M. Abtahian Z. Nasri E. (2018). Severe disseminated phaeohyphomycosis in a patient with inherited CARD9 deficiency. *Arch. Clin. Infect. Dis.* e84006. 10.5812/archcid.84006

[B30] Vinh D. C. (2011). Insights into human antifungal immunity from primary immunodeficiencies. *Lancet Infect. Dis.* 11 780–792. 10.1016/S1473-3099(11)70217-1 21958581

[B31] Wang X. Wang A. Wang X. Li R. Yu J. (2018a). Cutaneous mucormycosis caused by *Mucor irregularis* in a patient with CARD9 deficiency. *Br. J. Dermatol.* 10.1111/bjd.17144 [Epub ahead of print]. 30187457

[B32] Wang X. Zhang R. Wu W. Song Y. Wan Z. Han W. (2018b). Impaired specific antifungal immunity in CARD9-deficient patients with phaeohyphomycosis. *J. Invest. Dermatol.* 138 607–617. 10.1016/j.jid.2017.10.009 29080677

[B33] Wang X. Wang W. Lin Z. Wang X. Li T. Yu J. (2014). *CARD9* mutations linked to subcutaneous phaeohyphomycosis and TH17 cell deficiencies. *J. Allergy Clin. Immunol.* 133 905–908. 10.1016/j.jaci.2013.09.033 24231284

[B34] Yamamoto H. Nakamura Y. Sato K. Takahashi Y. Nomura T. Miyasaka T. (2014). Defect of CARD9 leads to impaired accumulation of gamma interferon-producing memory phenotype T cells in lungs and increased susceptibility to pulmonary infection with *Cryptococcus neoformans*. *Infect. Immun.* 82 1606–1615. 10.1128/IAI.01089-13 24470469 PMC3993402

[B35] Yan X. X. Yu C. P. Fu X. A. Bao F. F. Du D. H. Wang C. (2016). *CARD9* mutation linked to *Corynespora cassiicola* infection in a Chinese patient. *Br. J. Dermatol.* 174 176–179. 10.1111/bjd.14082 26440558

